# On general cognitive functioning. Descriptive study in post-intensive care syndrome patients after COVID-19 infection in a functional rehabilitation unit in Spain. A pilot study

**DOI:** 10.1192/j.eurpsy.2021.1765

**Published:** 2021-08-13

**Authors:** M.J. Maldonado-Belmonte, E. Fernández-Jiménez, J.M. Román-Belmonte

**Affiliations:** 1 Clinical Psychology, Hospital Central de la Cruz Roja, Madrid, Spain; 2 Idipaz, Department Of Psychiatry, Clinical Psychology And Mental Health, La Paz University Hospital, Madrid, Spain; 3 Rehabilitation, Hospital Central de la Cruz Roja, Madrid, Spain

**Keywords:** Post-Intensive Care Syndrome, Clinical Neuropsychology, COVID-19, Global cognitive functioning

## Abstract

**Introduction:**

Post-Intensive Care Syndrome (PICS) is a physical, cognitive, emotional and functional condition resulting from prolonged stays in ICU (Intensive Care Unit). In pathologies with clinical characteristics similar to SARS-CoV-2 pneumonia, most patients showed cognitive deficits after discharge from ICU. Further studies are needed to explore global cognitive impairment among PICS patients after COVID-19 infection.

**Objectives:**

To analyse the global cognitive functioning in patients with PICS after COVID-19 infection in a Functional Rehabilitation Unit in Madrid (Spain) using the Spanish version of the Screen for Cognitive Impairment in Psychiatry (SCIP-S).

**Methods:**

This study was conducted in the Hospital Central de la Cruz Roja, in Madrid (Spain). A sample of 17 PICS adult patients was included, with age ranging from 56 to 74 years old (mean = 68.35 years; 13 males). Patients were assessed around three weeks after referral from their reference hospital. The total score of the SCIP-S was used as outcome. Descriptive analyses were conducted (mean and standard deviation) on standardized scores (z) based on age-adjusted general population norms. Significant impairment was set at z < -1.5.

**Results:**

Mean total z-score on SCIP-S was -1.08 (S.D. = .82) from the total sample, with 52.9% of cases with significant impairment (mean = -1.74; S.D. = .21).

**Conclusions:**

These preliminary results show the probable presence of mild-moderate global cognitive impairment in a relevant proportion of patients after COVID-19 infection. Longitudinal studies, with larger samples, are needed where the premorbid cognitive level is considered.

**Disclosure:**

No significant relationships.

